# Supplementary material for: Are carbon emissions trading and green financial instruments synergistic? -Comprehensive quantitative research based on content analysis
Source: PLoS One. 2024 Mar 7;19(3):e0298601. doi: 10.1371/journal.pone.0298601 (PMC10919601; doi:10.1371/journal.pone.0298601)
Supplement: S1 Data — (ZIP) [file pone.0298601.s001.zip › Data/地级市Z_F工作报告.zip!/╔┬╬≈/╙▄┴╓╩╨/└┤╫╘╣1⁄2╓┌║┼í╛┬φ┐╦╩2╛▌═°í┐_2014.docx]

各位代表：

　　现在，我代表市人民政府向大会作工作报告，请予审议，并请市政协委员和其他列席人员提出意见。一、 2013 年工作回顾

　　2013 年，是进入新世纪以来我市经济发展最为困难的一年。面对煤炭价格下滑、投资增长放缓和民间借贷危机等一系列前所未有的严峻考验，我们全面贯彻落实中省和市委的决策部署，围绕市三届人大四次会议确定的目标任务，迎难而上，综合施策，全市经济社会发展稳中有进、稳中向好。全市完成生产总值 2846.8 亿元，按可比价计算，增长 8.8% ；全社会固定资产投资 1827.9 亿元，增长 3.2% ；财政总收入 668.9 亿元，其中地方财政收入 260.7 亿元，增长 19.6% ；社会消费品零售总额 298.6 亿元，增长 10.5% 。城乡居民收入分别达到 26820 元、 8687 元，增长 11.1% 、 13.1% 。特别是我们做了一些打基础、利长远的大事、难事和实事：一是兑现了 “ 三年缓解城市交通拥堵 ” 的承诺，榆林城区累计建成主次干道 47 条 78.3 公里、大型桥梁 6 座，打通了一批 “ 断头路 ” ，城区交通拥堵状况基本解决；二是兑现了 “ 三年缓解城区上学难 ” 的承诺，榆林城区累计开工建设学校 45 所，上学难问题基本解决；三是彻底关闭了备受关注的中能榆阳煤矿，消除了对榆林城市发展的影响；四是强力推进了中煤甲醇醋酸系列深加工及综合利用、靖边能化园区等一批投资超百亿元的重大转化项目，为转型升级积蓄了后劲。

　　一年来，我们主要做了以下工作：

　　（一）全力以赴稳增长，经济在下行压力和困难中保持平稳运行。出台了稳工业十项调控措施，工业经济企稳向好，原煤产量达到 3.39 亿吨，增长 4.8% ；原油 1230 万吨，增长 5.9% ；原油加工量 386 万吨，增长 14.1% ；天然气 150 亿立方米，增长 16.8% ；原盐 112 万吨，增长 6.8% ；发电量 476 亿度，增长 7.4% ；兰炭 2237 万吨，增长 14.8% ；金属镁 33 万吨，增长 8.4% ；电石 167 万吨，增长 14.4% ；精甲醇 158 万吨，增长 19.3% ；实现规模以上工业增加值 1901 亿元，增长 9.6% 。不断健全项目包抓包联和考核督查机制，积极破解制约因素，着力解决阻工问题， 100 个重点项目完成投资 606.9 亿元，小纪汗煤矿等 22 个项目建成或基本建成。通过设立 2 亿元专项企业扶持资金、召开银企对接会、推动民企与国企深度合作等措施，帮助非公企业渡难关，非公经济实现持续增长，增加值达到 1107 亿元，占全市生产总值比重达到 38.9% ，比 2012 年提高了 1.3 个百分点。

　　（二）积极主动调结构，经济发展质量和效益进一步提升。出台了全市现代产业体系等一系列规划，为有序推进产业转型升级明确了方向和任务。一批重大转化项目逆势而上，陕西有色榆林铝镁合金等 6 个资源转化项目建成投运。工业园区建设迈出新步伐，榆神工业区升级为国家级经济技术开发区，成为国家新型工业化示范基地。煤炭、兰炭产业整合提升取得阶段性成果，全市地方煤矿整合为 230 处，单井规模达到 97 万吨 / 年； 44 个涉及兰炭项目整合为 10 个综合利用项目，淘汰落后产能 955 万吨。新能源产业迈上新台阶，靖边建成全省首个 “ 百万千瓦新能源示范县 ” 。传统轻纺产业稳步振兴，羊毛防寒服产销额占到全国的 75% 以上，获得 “ 中国羊毛防寒服名城 ” 称号。现代特色农业再结硕果，粮食总产量达到 154.8 万吨，实现 “ 十连丰 ” ；榆林现代农业科技示范区升级为国家级农业科技示范园区；认定省级现代农业园区 34 个，发展家庭农场 2.6 万个，流转土地 162 万亩，均居全省第一。旅游文化产业取得新突破，融资注册 14 亿元的榆文旅公司挂牌成立；神木石峁遗址入选世界十大田野考古发现；全市接待游客 1470 万人次，实现旅游综合收入 75 亿元，分别增长 25.6% 、 28.2% 。科技创新成效显著，榆林国家创新型城市试点报批工作基本完成，世界首个华电 “ 循环硫化床煤制芳烃技术 ” 、全球首创的神木富油 “ 中低温煤焦油全馏分加氢技术 ” 通过中国石化联合会科技成果鉴定，神木兰炭与捣固焦配合用于高炉炼铁技术达到国际先进水平，我国首套规模最大的催化裂解制乙烯装置封顶。

　　（三）多措并举抓统筹，城镇化建设取得新突破。中心城区框架进一步拉大， “ 一城五区 ” 格局初步形成；建榆路、榆阳河大桥等 28 个路桥项目建成通车；榆溪河生态长廊提升改造工程顺利推进，东沙生态公园一期工程竣工，榆林大道、环城北路等 13 条道路绿化全面完成； “ 四城联创 ” 全面推进，获得 “ 省级文明城市 ” 称号，省级环保模范城市通过验收， “ 智慧榆林 ” 和 “ 智慧城管 ” 启动建设，榆林城市 “ 一卡通 ” 与全国联网。城乡一体化发展步伐加快，《榆林市城乡一体化建设规划（ 2013—2030 年）》编制完成；横山撤县设区、神木撤县设市工作上报国务院待批；锦界、东坑两个省级重点示范镇建设完成投资 11.8 亿元， 12 个市级重点示范镇和 12 个市级新型农村示范社区建设全面展开；全市城镇化率达到 52.8% 。区域经济协调发展，省市帮扶、北部县区对口帮扶、企业援助、人才帮扶的 “ 四位一体 ” 振南模式进一步完善，振南项目总投资达到 10.2 亿元。

　　（四）加大投入惠民生，老百姓享受到更多发展成果。在财政减收压力大的情况下， “ 两个 80%” 得到全面落实，民生支出达 360 亿元。教育事业优先发展，全市实施了 “15 年免费教育 ” ， 52.6 万名学生受益；榆林城区高新二小、市一中等 10 所新建学校建成投用；榆阳、神木、靖边通过 “ 双高双普 ” 验收，府谷建成省级教育强县。医疗卫生事业加快发展，市一院二期工程主体封顶，市二院装修工程全面推开，市三院开工建设；县级公立医院综合改革深入推进，基层医疗卫生机构综合改革进一步巩固完善，药品价格平均下降 30% ，医疗服务县镇一体化改革全面启动。劳动就业形势稳定，城镇新增就业 2.7 万人；转移农村劳动力 65.3 万人，创收 71.2 亿元。社会保障水平进一步提升，新农合、城乡居民养老保险、城乡低保、城镇职工退休养老金和工伤保险 7 项标准全部高于全省水平，其中新农合筹资标准每人每年提高到 415 元，城乡居民养老保险都提高到 1260 元，城乡低保分别提高到 4800 元、 2320 元。保障房建设稳步推进，新开工各类保障房 29434 套。扶贫开发力度加大，实施移民搬迁 7571 户、 28570 人， 10.7 万贫困人口实现脱贫。抗洪救灾和灾后重建扎实开展，妥善安置受灾群众，确保每一户受灾群众安全温暖过冬。平安榆林创建深入推进，安全生产形势稳定，组织开展了信访 “ 三百 ” 活动，在全市推行了社会治安 “ 四色分级 ” 预警机制，社会治安满意率全省排名提升了 2 个位次。

　　（五）下大力气强基础，瓶颈制约逐步缓解。交通体系日趋完善，榆佳高速建成通车，神佳米高速（神佳段）开工建设；沿黄公路一期工程路基全线贯通，定边过境公路建成通车。水源工程建设加快推进，王圪堵水库实现低水位通水，绥德、吴堡、佳县、子洲 4 个县城供水管网改造顺利实施；新建农村安全饮水供水工程 587 处，解决了 30 万人的饮水困难问题。电力保障更加有力，榆林首条 750 千伏电厂上网线路并网运行。生态环境持续改善，完成绿化造林 105.5 万亩；治理水土流失 1216.5 平方公里。节能减排扎实有效，全省排污总量预算和排污权交易试点启动，榆横工业区污水处理厂建成投用，神木、府谷等 7 个污水处理厂提标改造完成，榆林城区、各县城垃圾处理率分别提高到 89.3% 和 60% ，污水处理率分别提高到 73.8% 和 69% 。榆林城区好于二级以上天数达到 322 天，其中一级天数创有监测记录以来最好水平，达到 99 天，同比增加 49 天。

　　（六）坚定不移促改革，发展活力明显增强。重点领域改革取得积极进展，我市成为省级综合配套改革试验区，省级金融综合改革试点市建设启动，府谷县省级民营经济转型升级试验区建设有序推进，食品药品监管改革工作顺利完成。国库集中支付改革不断深化，全市 2873 个预算单位纳入国库集中支付改革范围，覆盖率达到 89% ；市县公务卡制度改革全面启动。招商引资数量质量双提升，实施招商引资项目 280 个，资金到位 603.5 亿元，其中非能化类项目占到 80% 。金融市场保持基本稳定，全市新开业农村商业银行 4 家，新增保险机构 3 家；采取区别对待、分类处置、打调结合的办法，有力地打击了非法集资，民间融资风险得到有效控制。政府自身建设成效明显， “ 八项规定 ” 得到有效落实，会议、文件数量大幅压缩， “ 三公 ” 经费下降 15% ；开展了 8 次公民代表走进市政府活动；县区、乡镇便民服务中心实现全覆盖，村（社区）便民服务室覆盖率达到 60% ，办理便民服务事项 59.8 万件；自觉接受人大、政协、人民群众和新闻媒体的监督，办理人大代表建议 117 件、政协委员提案 335 件，办复率均达到 100% 。

　　一年来，司法、物价、计生、体育、统计、审计、工商、质监、人防、供销、气象、扶残助残、民族宗教、国防和军队建设等都取得了新成绩。

　　各位代表，过去的一年，我们以咬定青山不放松的韧劲，既牢牢抓好项目推进、民生改善和城市建设等当前发展中的重点工作，又积极破解产业升级、生态建设和深化改革等事关榆林长远发展的重大问题，做了许多艰苦工作，取得一定成绩。经济增长与预期目标虽有一定差距，但仍处于合理增长区间。可以说，挑战非同寻常，过程充满艰辛，成绩来之不易。这是市委把握大局、正确领导的结果，是市级几套班子和衷共济、团结奋进的结果，更是全市人民艰苦努力、共同奋斗的结果。在此，我代表市人民政府，向全市广大干部群众、驻榆部队指战员、武警消防官兵、公安民警致以崇高的敬意！向大力支持政府工作的各位人大代表、政协委员和社会各界人士表示衷心的感谢！

　　在总结成绩的同时，我们更应清醒地认识到，榆林仍然是一个 “ 经济发展较快的落后地区 ” ，发展中不平衡、不协调、不可持续的问题依然突出：产业结构不合理，科技创新能力不强，发展方式粗放，推动转型升级任重道远；市场主体活力仍显不足，投资拉动增长难度增加；民间融资信用体系遭受重创，部分民营企业流动资金紧张，生产经营出现困难；城乡区域发展和居民收入差距依然较大，统筹发展的任务艰巨；基础设施承载能力仍然不足，资源环境约束日益趋紧；创新社会治理工作亟待加强，公共服务能力需要不断提高； “ 四风 ” 问题还不同程度存在，干部作风和政府自身建设有待进一步加强。对此，我们必须采取有力措施，切实加以解决。

　　二、 2014 年工作的指导思想和目标任务

　　2014 年，是贯彻落实党的十八届三中全会精神、全面深化改革的重要一年，也是完成 “ 十二五 ” 规划的关键一年。从外部环境看，世界经济结构进入调整期，经济治理机制进入变革期；我国经济进入增长速度换挡期、结构调整阵痛期和前期刺激政策消化期。我们将认真分析和准确把握国内外经济发展的新趋势，努力提高应对复杂局面的能力，抢占未来发展的战略制高点。从自身发展看，受外部宏观环境传导效应影响，煤炭价格回升的迹象不明显，我市经济形势依然严峻、下行压力依然较大，但经过多年努力，全市进入 “ 四化 ” 同步发展的新阶段，一批拥有世界领先技术投资超百亿元的重大项目建设，为推动经济持续发展注入新动力，一批重大基础设施项目显著提升了发展保障能力。从政策层面看，党中央对全面深化改革作出总体部署，我市上升到《全国资源型城市可持续发展规划》等 7 个国家级规划发展战略，省委、省政府出台了进一步支持榆林持续发展的新 “27 条 ” 政策，这些利好因素将有效激发我市发展潜力。我们完全有条件、有能力保持经济合理增长、质量效益稳步提升。

　　今年政府工作的指导思想是：以科学发展观为指导，全面贯彻落实党的十八大、十八届三中全会精神，按照中央经济工作会议、省委十二届四次全会和市委三届三次全会部署，牢牢把握稳中求进的工作总基调，在稳定增长中优化结构，在深化改革中创新发展，在扩大内需中推进开放，在改善民生中促进和谐，全力打造榆林经济升级版，加快全面建成小康社会进程。

　　全市经济社会发展的主要预期目标是：生产总值增长 10% 左右；全社会固定资产投资增长 15% ；地方财政收入增长 13% ；城乡居民收入分别增长 11% 、 12% ，社会消费品零售总额增长 14.6% ，居民消费品价格指数涨幅控制在 4% 以内，人口自然增长率控制在 6.5‰ 以内，城镇登记失业率控制在 4% 以内。今年经济预期增长目标，既是积极有为的体现，又充分考虑了各种有利不利因素；既顺应了我市经济持续增长趋势，又与中长期发展目标相衔接；既可为转型升级赢得时间和空间，又能为保障和改善民生创造必要条件。

　　实现以上目标任务，我们将牢牢抓住以下五个着力点：

　　一是抓改革，促红利释放。把深化改革作为稳中求进的根本动力，将改革创新贯穿于经济社会发展各个领域各个环节，坚持问题导向，区分情况，分类推进，努力让一切创造社会财富的源泉充分涌流。有序承接上级改革政策，准确把握中央和省委、省政府关于全面深化改革的安排部署，研究贯彻方案，适时、有序、协调推进。强力推进本级改革部署，使市场在资源配置中起决定性作用和更好发挥政府作用有机结合起来，加快推进方向明、见效快、可操作的改革，稳扎稳打，着力在教育卫生、民营经济、财政金融、生态文明等重要领域和关键环节取得积极进展。

　　二是抓项目，促转型升级。把主动转型作为稳中求进的重中之重，充分发挥投资的关键作用，着力围绕煤炭做转化、围绕转化做规模、围绕规模深加工，加快培育具有榆林特色的现代产业体系，打造能源化工基地升级版。优化投资，促进投资合理增长和结构优化，强势推进超百亿元重大能化项目建设，谋划招引一批事关全市转型发展的高端化工项目。毫不动摇鼓励、支持、引导非公经济发展，坚持权利平等、机会平等、规则平等，发掘民间投资潜力，激发非公经济活力和创造力。优化产业，推广煤基多联产模式，构建以煤炭中低温干馏推动煤制烯烃、煤制芳烃、煤制油、煤盐一体化发展的 “ 一推四 ” 特色煤化工产业体系；大力发展现代特色农业，优化升级羊毛防寒服等传统产业；培育发展太阳能、风能、文化旅游、能化装备制造和配套物流等接续产业，形成多点支撑、多元带动的新格局。

　　三是抓城镇，促统筹发展。把城镇带动作为稳中求进的最大潜力，积极稳妥推进新型城镇化，加快构建以城带乡、城乡一体、南北协调的新型城乡区域关系。提升以人为核心的城镇化水平，优化中心城市空间布局，加快 “ 一城五区 ” 重点区块开发，完善服务功能，彰显特色魅力，加快建设区域中心城市；提升县城建设品位，引导优势产业向县城集聚；发展有历史记忆、地域特色的美丽城镇，促进中心城区、县城和小城镇合理分工、协同发展。提升区域发展的协调性，坚定不移实施主体功能区规划，推动自然条件不同区域按不同定位发展；完善区域发展一体化体制机制，推动公共服务资源均衡配置。

　　四是抓民生，促社会和谐。把改善民生作为稳中求进的根本目的，切实改善民生，创新社会治理，促进社会既充满活力又和谐有序，让人民群众切身感受到幸福榆林的实惠。突出民生优先，按照保住基本、突出重点、完善制度的思路，严格落实 “ 两个 80%” 硬指标，统筹做好就业、社保、教育、卫生、住房等改善民生各项工作，着力解决好群众最关心、最直接、最现实的利益问题。创新社会治理，及时协调人民群众各方面、各层次利益诉求，弘扬社会主义核心价值观，加快建设诚信榆林；健全公共安全体系，全面推进平安榆林建设，最大限度增加和谐因素。

五是抓生态，促环境改善。把优化生态作为稳中求进的重要保障，坚持绿色发展、循环发展、低碳发展，尽可能减少对自然的干扰和损害，加快建设人与自然和谐发展的美丽榆林。用制度完善保护生态环境，加快建立系统完整的生态文明制度体系，划定生态保护红线，实行最严格的源头保护制度、损害赔偿制度、责任追究制度，完善环境治理和生态修复制度。用绿色经济优化生态环境，加快建设国家级循环经济试点市，将生态效益和经济效益相结合，推进资源循环式利用、产业循环式耦合、区域循环式开发，促进生产空间集约高效、生活空间宜居舒适、生态空间山清水秀。

三、 2014 年重点工作

　　围绕上述指导思想和目标任务，今年重点抓好以下八项工作：

　　（一）创新引领，主动转型，打造榆林经济升级版。

　　促进能化经济提质增效升级。全力以赴推动 15 个投资超 100 亿元重大能化项目建设，支持神华锦界电厂等 2 个建成项目做大做强，确保中煤甲醇醋酸系列深加工及综合利用等 5 个续建项目早日达产达效，开工建设华电 100 万吨煤制芳烃等 3 个项目，做好华能段寨煤电一体化等 5 个项目前期工作。以销促产做大煤炭采掘业，全面建成金鸡滩等 16 个煤矿和西岔沟等 36 个整合煤矿，依法有序释放浅层煤和边角煤产能；积极帮助企业做好煤炭销售工作，支持陕汽、陕煤化、榆能等建设榆林至安康煤炭外运绿色通道，促进发电企业与用户直接交易。加快推进勘探开发，推广新技术新工艺提高油气采收率，增强原油就地转化能力。提升兰炭、电石、金属镁、铝镁合金等本土特色载能产业。全年生产原煤 3.75 亿吨、原油 1265 万吨、原油加工量 420 万吨、天然气 165 亿立方米、发电量 500 亿度、兰炭 2300 万吨、精甲醇 210 万吨、醋酸 10 万吨、聚氯乙烯 100 万吨、金属镁 42 万吨、铝镁合金 40 万吨、电石 180 万吨。

　　积极调整工业产业结构。加快榆林国家级新能源示范城市创建工作，推进李家梁风电二期等 3 个续建项目建设，启动建设榆神协和光伏电站等 9 个项目，新增新能源装机容量 430 兆瓦。开工建设榆神工业区西部机电设备城，支持陕汽榆林东方新能源专用车、陕西神木新大通专用车等企业加快发展。加大 “ 中国羊毛防寒服名城 ” 宣传力度，打造羊毛防寒服交易中心。加快发展红枣、杂粮、羊肉等农副产品和黄芪、沙棘等中药材加工业。大力发展蓖麻、长柄扁桃等生物质提取，延长产业链条。认定 10 户转型升级示范企业。

　　发展壮大现代服务业。制定全市旅游发展总体规划，促进特色文化与旅游业融合发展，打造精品旅游线路；提升白云山、红碱淖等景区发展水平；支持榆文旅公司开发榆林古城、统万城、东方红纪念园等重点景区；加快杨家沟、波罗古堡、府州古城、杨家城、黄土风情园、毛泽东诗词园等重点景区建设；加大神木石峁遗址、靖边丹霞地貌等旅游资源开发力度。开拍电视剧《平凡的世界》，推出《大漠红柳》《我的三十里铺》等更多文化精品。大力发展现代物流业，加快建设榆林陆路货运口岸。精心组织各种消费促进活动，继续开展万村千乡、农超对接、外贸孵化器和农贸市场改造工程，积极引进沃尔玛、红星美凯龙等国内外知名企业，培育壮大喜洋洋、国贸等本土企业。严格落实带薪年休假制度，大力培育网购、休闲、健康、养老、信用等消费业态。

　　提升科技创新能力。强化企业在技术创新中的主体地位，支持中省企业在榆建立实验室、研发中心。积极与科研院校合作，全力攻关面煤制兰炭技术难题，拓展兰炭在冶炼、供热等领域应用。加快制定 “ 榆林煤 ” 地方标准，申请注册 “ 榆神煤 ” 品牌。加大白绒山羊、马铃薯良种选育工作力度。加快信息化基础设施建设和信息公共资源整合，推进 “ 智慧城市 ” 和 “ 两化融合 ” 建设，构建统一的电子政务网络平台和政府综合门户网站群。

　　激发园区发展活力。进一步理顺园区发展体制机制，充分发挥大园区示范辐射带动作用，榆林高新区重点加快未开发区块建设步伐，完善工业配套设施，强力推进重点项目建设，完成生产总值 300 亿元；榆神工业区加快建设国家能源科技示范基地，重点推动重大能化项目及大保当镇后勤服务基地建设，完成生产总值 200 亿元。加大各县区省级重点工业园区建设，在基础设施、招商引资等方面取得较大进展。

　　（二）强基提质，多措并举，促进 “ 三农 ” 健康发展。

　　发展现代特色农业。严守耕地红线，粮食播种面积稳定在 710 万亩左右，着力打造陕西第二粮仓。加快推进农业产业化，做大做强玉米、马铃薯、小杂粮、蔬菜等主导产业，加快发展山地苹果产业；创新农业生产经营方式，有序推进现代农业园区、龙头企业、一村一品示范村、专业合作社、家庭农场 “500 强 ” 工程建设。推动畜牧业健康发展，培育畜禽规模养殖大户 3000 户、适度规模家庭农场 300 个，完成人工种草 100 万亩，羊子饲养规模稳定在 1000 万只、生猪 260 万头、家禽 1100 万只。实施品牌战略，启动榆林 “ 马铃薯、小杂粮、红枣、大漠蔬菜、山地苹果、羊肉 ” 六大区域公用母品牌商标申报注册工作。加大农民培训力度，培育新型职业农民。完善农产品市场体系，重点支持定边农产品物流交易中心、靖边东坑物流交易中心、米脂小杂粮交易中心、绥德裴家峁综合批发市场建设。

　　抓好农业生产保障能力建设。实施农田水利基本建设等项目，新建基本农田 7 万亩。抓好小流域坝系、新一期国家水土保持重点建设治理等工程，新建、加固、维修淤地坝 850 座，完成水土流失治理 1100 平方公里。开展无定河全线综合治理项目、黄河粗泥沙集中来源区拦沙工程前期工作。强化气象为农服务体系建设，适时开展人工增雨作业。普及推广农机新技术，扩大农业机械化生产规模。巩固农村安全饮水成果，新解决 25 万农村人口不安全饮水问题。实施农网升级改造工程，推进城乡供电一体化。改造县乡公路 200 公里，建成通村油（水泥）路 2500 公里，加强对建成通村公路的维护和管理。

　　扎扎实实打好扶贫攻坚战。全面实施吕梁山片区区域发展与扶贫攻坚规划，按照 “ 一村一策、一户一法 ” 精准扶贫的思路，推进移民搬迁、整村推进、产业开发、能力建设、外资扶贫、社会扶贫 “ 六大工程 ” ，实施整村推进项目 204 个、扶贫移民搬迁 6000 户 2 万人，全年脱贫 10 万人，让贫困地区群众生活不断好起来。

　　（三）项目带动，优化环境，拉动经济持续增长。

　　抓好重点项目建设。全市安排重点项目 105 个，总投资 3844 亿元，年度完成投资 580 亿元。全力推动神华甲醇下游加工等 68 个续建项目建设；开工建设郭家湾煤矿等 37 个项目，在 2014 年陕北能化基地座谈会期间集中开工一批重大项目；建成或基本建成靖边能化园区一期等 36 个项目；做好 60 个重大项目的前期工作；力争全年新入库 100 个符合国家投资方向和榆林转型升级的项目，不断增强发展后劲。

　　加强重大基础设施建设。铁路方面，建成准神铁路，完成神朔铁路万吨扩能改造，加快准朔铁路、榆横铁路二期、府谷煤炭铁路、小纪汗铁路专用线建设，力争开工建设靖边至神木铁路和孤山川至庙沟门、朱盖塔至大保当等支线铁路，做好蒙华铁路环境保障工作，搞好府兴铁路和神瓦铁路前期工作，争取西安至榆林特快、榆林至北京直达列车早日开通。公路方面，完成神佳米（神佳段）高速路基工程，加快推进绥清高速前期工作；完成大柳塔至石马川、店塔至红碱淖、神木至盘塘一级公路路基桥涵工程；建成清涧至辛关、府谷墙头至凤凰塔二级公路；开工建设 307 国道绥德西过境线、榆林绕城快速干道西过境线等项目，加快推进米脂、清涧、子洲、吴堡等县国省道过境公路建设；完成沿黄公路一期工程。机场方面，加快波罗通用机场建设，力争开工建设榆阳机场扩建工程、神木通用机场和府谷支线机场。水利方面，王圪堵水库实现供水，力争并网发电，加快推进榆神工业园区供水工程，加大中心城区和各县城水源及供水管网建设力度；开工建设榆佳工业园区供水工程、清涧延安延川支线引水单项工程、绥德枣林坪引水工程；加快推进黄河东线大泉引水前期工作。电力方面，开工建设 330 千伏府谷 2 号及定边输变电工程，建成和增容 110 千伏工程 20 站 43 线、 35 千伏工程 1 站。

　　优化项目建设环境。强化 “ 五个一 ” 工作机制，建立百亿元重大项目市县乡三级包抓班子，完善重点项目进展媒体公示、督查、奖惩等措施，实行项目定期调度制度，严格对项目复工率、开工率、投资率等进行考核评价。依法打击破坏重点项目建设的违法犯罪行为，强化环境保障问责，确保重点项目顺利推进。

　　（四）统筹兼顾，突出重点，推进新型城镇化进程。

　　加快中心城市建设。按照 “ 新区带动、板块推进、拉大框架、组团发展、提升品位 ” 的工作思路，积极推进榆横一体化，继续加大中心城市建设力度，实现 “ 三年大变样 ” 的目标。坚持一张蓝图干到底，启动中心城区空间战略发展规划，依法查处违反规划的建设行为。优化路网体系，榆林大道北段等 19 个续建项目建成通车，开工建设榆佳路改造等 24 个路桥项目，启动建设高新区榆靖高速互通立交、跨榆横铁路大桥和建业大道跨榆溪河大桥。推进片区开发，将榆林大道、东山大道、迎宾大道两侧和榆溪河两岸、中心商务区、高新区等核心区域进行项目打包，整体有序快速推进。完善公用设施，启动东、南、北 3 个汽车客运站建设，开通 3 条公交线路，投放一批出租车；建成榆溪河截污干管工程，实施梅花楼、城区景观亮化等改造工程，完成红山路西段、迎宾大道等排水排污工程，启动西沙 7 万吨水厂、高新区供水厂二期项目建设，硬化改造巷道 40 条，加大热源点建设，新建供热管网 20 公里，新增天然气用户 1 万户。按照政府主导、市场运作原则，稳步推进崇文雅苑等 7 个平房片区和流水沟村等 4 个城中村改造项目。全面落实人防工程建设前置审批，不断增强城市整体防护能力。提高城市精细化管理水平，加大国家级卫生城市、国家园林城市创建力度，启动国家节水型城市、省级生态园林城市创建工作，让人民群众生活得更加舒适、更加幸福。

　　增强县城和重点镇综合承载能力。促进产城融合发展，推进横山撤县设区和神木、靖边、绥德撤县设市工作，加快县城基础设施和公共服务设施建设，进一步提升管理水平。继续抓好锦界、东坑两个省级重点镇和高家堡、波罗、名州 3 个省级文化旅游名镇建设，扎实推动市级 “ 双十二 ” 试点建设，发挥示范带动效应。保留村庄原始风貌，慎砍树，少拆房，尽可能在原有村庄形态上，通过加大基础设施建设改善居民生活条件。完善城镇化配套政策体系，全面放开县城和建制镇的户籍限制，推进有能力在城镇稳定就业和生活的常住人口有序实现市民化，稳步提高户籍人口城镇化水平，完成 12 万有条件农村居民进城落户，全市城镇化率提高到 53.86% 。

　　促进南北协调发展。以转移南部人口为核心，以发展民生事业为重点，以改善基础设施为突破，以发展特色产业为支撑，加大振南工作力度。继续强化 “ 四位一体 ” 振南机制，加大市级支持力度，严格落实北部县区上年度地方财政收入的 1% 对口扶南的政策，积极落实中省企业对口帮扶政策，大力实施项目振南，增强南部县自我发展能力。推动北部县区转型升级，培育经济发展新动力。

　　（五）深化改革，扩大开放，增强发展内生动力。

　　推进重点领域改革。稳步推进矿产资源制度改革，加大招拍挂方式出让探矿权力度。探索建立城乡统一的建设用地市场，推进国土空间布局和土地利用优化试点。出台全市农村土地流转指导意见，推进农村承包地、宅基地、集体建设用地确权登记发证工作，探索建立农村产权流转交易市场，赋予农民更多财产权利。推进省级金融改革试点市建设，引导在榆金融机构加大对本土企业的支持力度，合理引导规范民间融资，支持符合条件的民间资本依法设立民营银行、金融租赁公司和消费金融公司等金融机构，多渠道推动股权、债券融资，提高直接融资比重。加强社会信用体系建设，启动创建全国信用体系试点市。制定能化基地水资源管理办法，理顺水政管理机制。全面实施工商登记制度改革，削减资质认定和前置审批项目，由 “ 先证后照 ” 改为 “ 先照后证 ” ，把注册资本实缴登记制改为认缴登记制。推进公立医院综合改革，在全市所有乡镇卫生院实行 “ 先住院、后结算 ” ，在 1—2 个县级医院开展 “ 先住院、后结算 ” 试点。支持和推进神木、府谷省直管县改革试点工作，赋予神府两县市级经济管理权限，加快神府经济转型升级步伐。

　　支持非公经济健康发展。全面落实支持民营经济发展的有关政策，提振民营企业发展信心，实现 “ 二次创业 ” 。大力发展混合所有制经济，力争陕煤化、延长石油、陕有色等国企与我市重点民营企业合作取得实质性进展。加快民营经济转型升级，鼓励和引导民营企业延伸产业链，投资新兴产业；鼓励支持有条件的民营企业建立现代企业制度。加大财政支持力度，通过 “ 股权投资 ” ，开辟支持民营企业发展新途径，筹集 1 亿元扶持民营经济发展资金用于 “ 助保贷 ” ，帮助民营企业缓解融资难题。深入推进府谷省级民营经济转型升级试验区建设。非公经济增加值占生产总值的比重达到 40% ，新增从业人员 1 万人。

　　全面创新招商方式。围绕精细化工、聚氯乙烯加工、金属镁加工、轻纺、农特产品加工、文化旅游等领域，广泛组织开展以商招商、专业招商、定向招商、集群化招商、产业链招商，形成政府推动、园区带动、企业主导、中介服务的招商引资新格局，提升招商引资质量，加快项目落地进程。制定出台对中介组织、中介人等招商引资奖励办法，强化招商引资考核，形成全民招商的浓厚氛围。

　　（六）统筹安排，完善机制，织好保障民生安全网。

　　把就业工作摆到突出位置。全面落实在榆投资大中型企业当地用工比例不低于 60% 的就业政策，结合产业升级开发更多适合高校毕业生的就业岗位。进一步开发公益性岗位，通过向用人单位发放社会保险补贴、岗前培训补贴等办法，鼓励企业吸纳待业青年就业。完善扶持创业优惠政策，积极开展省级创业型城市创建工作，促进高校毕业生、农村转移劳动力、城镇困难人员、退役军人有效就业创业，营造全社会创业的浓厚氛围。全年新增城镇就业 2.7 万人，下岗失业人员再就业 7000 人，实现农村劳动力转移就业 65 万人。

　　建立更加公平可持续的社保体系。跟进中省政策，继续推进养老、医疗、工伤、生育、失业保险提标扩面工作。做好医疗保险市级统筹，平衡南北县区待遇差距。逐步推进省内异地就医结算。健全重特大疾病医疗保险和救助制度。启动以社区为依托的 “ 居家养老 ” 试点工作。不断完善城乡低保、五保供养、临时救助、流浪乞讨管理制度，实现特困人群救助全覆盖。健全农村留守儿童、妇女、老年人、残疾人关爱服务体系。新开工建设各类保障性住房 11250 套，推进廉租房与公共租赁住房并轨，公平合理分配已建成的保障房；加强房地产市场监管。强化食品药品监管，将农产品质量安全纳入县乡政府绩效考核，深入实施 “ 放心粮油 ” 全覆盖工程，建设 4 个市级农产品质量安全监管示范县，确保 “ 舌尖上的安全 ” 。

　　办好人民满意的教育。深入实施 “15 年免费教育 ” ，健全家庭经济困难学生资助体系。全面推进城区 26 个在建学校项目建设，高新中学等 18 个学校项目工程竣工，彻底解决榆林城区上学难、班额大问题。大力普及学前教育，启动新一轮学前教育 “ 三年行动计划 ” 。开展标准化学校建设工作，促进义务教育均衡发展，府谷建成义务教育均衡发展县，定边建成 “ 双高双普 ” 县。推动普通高中特色化发展，绥德中学建成省级示范高中。大力发展职业教育，建设 1 个现代化农业职教发展工程示范县，支持创建 1 个国家级农村职成教示范县。提升高等教育水平，积极推进榆林学院创办榆林大学工作，启动榆林职业技术学院二期工程建设。加强教育教学管理创新，强化教师队伍建设，培养一批 “ 名校长、名教师 ” ，促进教育内涵式发展，全面提高教育质量。

　　大力发展文体事业。建设榆溪河陕北民俗文化雕塑长廊，开工建设榆林文化艺术中心，完成统万城维修二期等 5 个文物保护工程。推动公共文化服务社会化发展，鼓励社会力量、社会资本参与公共文化服务体系建设，培育文化非营利组织，全面开展群众性文化活动。不断完善公共体育设施，开工建设榆林体育中心，广泛开展全民健身活动。

　　全面提升医疗卫生水平。推动医院标准化建设，加强内涵管理，提升服务质量，实现市县乡三级医疗机构差异化发展。市二院实现整体搬迁并正式投运，加快推进市一院、三院、星元医院、榆阳人民医院项目建设，启动建设高新区妇幼保健医院等 3 个专科医院，支持每个县创建一所二甲医院。切实做好医疗卫生人才培养和引进工作。落实好 “ 单独二孩 ” 生育政策，加大对计生家庭的奖励扶助力度，关爱失独家庭。

　　深入推进平安榆林建设。严格落实信访责任，设立各级矛盾调处中心，深入开展积案大清理活动；把涉法涉诉信访纳入法治渠道解决，建立涉法涉诉信访依法终结制度。强化安全生产责任，着力推进政府监管网格化、企业管理精细化、行业直管专业化，大力开展重点行业领域专项整治，坚决遏制重特大安全事故发生。加大公安科技信息化建设力度，创新公安战斗力生成模式，构筑立体化社会治安防控体系，严密防范、依法打击各类违法犯罪活动，切实维护社会稳定。完善网络监管机制，形成正面引导和依法管理相结合的网络舆论工作格局，凝聚社会正能量。健全应急管理机制，提高应急处置能力。

　　（七）加大投入，健全制度，着力建设美丽榆林。

　　持续加强生态建设。控制开发强度，规范开发秩序，严格落实企业生态治理责任。以 “ 三年植绿大行动 ” 为载体，依托三北防护林、京津风沙源二期治理等国家项目，稳步推进环榆林城防护林带等生态林业 “ 八大工程 ” ，狠抓樟子松等 “ 五个百万亩基地 ” 建设，完成植树造林 100 万亩，扩大绿色生态空间比重。实施好红碱淖国家级湖泊生态环境保护项目。继续抓好退耕还林工作，严格封山禁牧，强化森林防火，狠抓病虫害防治，巩固植树造林成果。

　　加大城乡绿化力度。按照尊重自然、顺应自然、天人合一的理念，依托现有山水脉络等独特风光，让城市融入大自然。中心城区依照 “ 城在林中、人在景中 ” 的思路，高标准绿化榆阳西路、建榆南路等新建道路，改造提升榆林大道、文化北路两侧绿化带和榆溪河滨河公园、东沙生态公园一期绿化景观，开工建设大墩梁森林公园一期工程。市财政在 “ 三年植绿大行动 ” 专项资金中列出部分资金用于县城绿化，依照 “ 有规划、有文化、本土化、园林化 ” 的要求，着力提升县城绿化水平。加强公路两侧绿化，搞好村镇绿化，形成城乡联动、全民造林绿化的社会新风尚。

　　扎实推进环境治理。严格实行建设项目环境总量指标前置审核，坚决落实 “ 一票否决 ” 、行政问责制，各工业园区污水处理厂和垃圾处理场建设不到位的，暂停项目审批。推进排污权交易试点，争取开展碳排放交易试点。推进水泥、石化、煤化工等行业二氧化硫和氮氧化物治理，减少主要污染物排放总量。启动榆林城区 4 个点位 PM2.5 监测工作，及时向社会发布监测数据。完善落后产能退出机制，加快小火电、小兰炭等落后产能淘汰，推动重点行业、产业提升技术水平。按照 “ 先安置、后开采 ” 的要求，加大采空塌陷区治理。做好水源地保护工作，确保饮用水水源地 100% 达标。围绕 “ 清洁家园、清洁田园、清洁水源、清洁能源 ” 四大目标，开展 “ 以奖促治、以创促治、以减促治 ” 三大整治活动，全面改善农村环境，推进美丽乡村建设工作。

　　（八）转变作风，提高效能，强化政府自身建设。

　　深化行政体制改革，增强推动发展的创造力。深化行政审批 “ 两集中、两到位 ” 改革，进一步简政放权，严格控制新设许可，规范非许可审批项目；加快市政府政务服务中心建设，完善市县乡三级便民服务体系。改革工程招投标管理办法，建立统一的招投标监管机构和交易平台，今年所有政府投资项目实行招投标摇号制度。大力推进事业单位人事管理制度改革。稳步推进公车改革，积极推行政府后勤市场化服务。

　　全面推进依法行政，增强干事创业的凝聚力。自觉接受人大依法监督、政协民主监督和社会舆论监督，团结各方力量推动工作。加强对政府决定事项和规范性文件的合法性审查，充分发挥行政复议在化解矛盾中的作用。深化政务公开，将部门预算和 “ 三公 ” 经费公开扩大到乡镇，提高财政资金管理水平；扩大公民代表走进政府和大学生到政府机关见习活动的范围；开通市县两级政务微博，加大群众网上诉求办理力度。落实执法人员持证上岗和资格管理制度，下决心解决粗暴执法问题，真正做到严格规范公正文明执法。支持监察、审计部门依法实施行政监督，实现审计监督全覆盖。

　　扎实开展群众路线教育实践活动，增强狠抓落实的执行力。

　　以 “ 为民、务实、清廉 ” 为主题，按照 “ 照镜子、正衣冠、洗洗澡、治治病 ” 的总体要求，扎实开展党的群众路线教育实践活动，认真解决 “ 四风 ” 和群众反映强烈的突出问题。严格执行 “ 八项规定 ” ，切实改进会风文风，除 “ 两会 ” 外，各级各类会议一律不得在宾馆酒店召开；加强调查研究，察实情、出实招、办实事，总结基层经验，推动工作落实。始终保持干事创业的热情、奋发有为的激情，以踏石留印、抓铁有痕的决心，不断创造经得起群众和历史检验的业绩。强化行政问责和效能监察，启动实施末位查究制，着力整治公务人员庸懒散浮等问题，提高政府系统执行力和工作推动力。

　　加大从严治政力度，增强廉洁从政的公信力。认真落实党风廉政建设责任制，推行部门权力清单制度，明确权力运行流程，加强动态预警，强化制约措施。严格政府举债程序，将政府性债务分门别类纳入全口径预算管理，强化债务考核，着力防控各级政府债务风险。坚决降低行政成本，进一步压缩财政性一般经费支出， “ 三公 ” 经费下降 10% 以上；坚决叫停新建楼堂馆所，全面清理党政机关和领导干部办公用房；超配车辆一律收回统一拍卖。坚持政府 “ 阳光采购 ” ，启用电子化采购平台，把有限的资金用在群众最关心、最需要解决的问题上。坚决查处各种违纪违法行为，以清正廉洁的良好形象取信于民。

　　各位代表！梦想承载希望，奋斗开创未来。让我们更加紧密地团结在以习近平同志为总书记的党中央周围，在省委、省政府和市委的坚强领导下，团结依靠全市人民，胸怀中国梦，积聚正能量，低调务实不张扬，埋头苦干，敢于担当，锐意进取，谱写建设幸福榆林的新篇章！
